# Characterization of Hashimoto´s thyroiditis in Sudanese children: a cross-sectional study at Gaafar Ibnauf Hospital, Khartoum

**DOI:** 10.11604/pamj.2023.44.86.35649

**Published:** 2023-02-14

**Authors:** Ghassan Faisal Fadlalbari, Salwa Abdelbagi Musa, Samar Sabir Hassan, Areej Ahmed Ibrahim, Mohamed Ahmed Abdullah

**Affiliations:** 1 Gaafar Ibnauf Pediatric Tertiary Hospital, Pediatric Endocrinology Unit, Khartoum, Sudan; 2 Department of Pediatrics and Child Health, Faculty of Medicine, University of Khartoum, Khartoum, Sudan

**Keywords:** Children, clinical features, Hashimoto´s thyroiditis, outcome, Sudan

## Abstract

**Introduction:**

literature on Hashimoto´s thyroiditis, the common thyroid illness in the young populations, in Sudan and Africa is scarce. We aimed to study its clinical profile and outcome among Sudanese children and adolescents.

**Methods:**

records of 73 patients were reviewed. Data related to demographics, presenting features, family history and coexistence of autoimmune diseases, physical examination findings, and biochemical progression over time were obtained.

**Results:**

patients´ mean age at the diagnosis was 10.6 ± 2.9 years, 80.8% (n = 59) of them were female and 83.6% (n = 61) were residing in iodine-sufficient areas. The commonest presenting features were thyromegaly and fatigability (79.5%, n = 58 and 43.8%, n = 32, respectively) after an illness duration of 0.5-48 months. Autoimmune comorbidities were documented in 8.2% (n = 6) of our series and more than half (53.4%, n = 39) of them were pre-pubertal at the diagnosis. Sixty point three percent (60.3%) (n = 44), 20.5% (n = 15), 13.7% (n = 10) and 5.5% (n = 4) of patients presented with overt hypothyroidism, sub-clinical hypothyroidism, euthyroidism and hyperthyroidism respectively, and there were no significant differences in the clinical profile between them. In patients’ continued follow-up, 94.1% (n = 32/34) of those presented with overt hypothyroidism required levothyroxine therapy to maintain euthyroidism for 0.5-13 years, while 85.7% (n = 6/7) of those with euthyroidism remained so for 0.5-6 years. Remission was reported in all hyperthyroid patients and in only 5.9% (n = 2/34) of those with overt hypothyroidism at diagnosis. The majority of our patients with subclinical hypothyroidism were treated with levothyroxine and continued to be euthyroid for 10 months to 13 years.

**Conclusion:**

goiter was the commonest presenting feature of Hashimoto´s thyroiditis. The majority of patients had overt or subclinical hypothyroidism and almost all of them required long-term levothyroxine therapy.

## Introduction

Hashimoto´s thyroiditis is a common acquired thyroid disorder, particularly in iodine-sufficient areas (ISA), with a prevalence of 1-3% in children [1-5]. Recently, it showed a rising incidence which has been referred to the advancement in thyroid autoantibody testing technology and availability of fine needle aspiration facilities [2].

Hashimoto´s thyroiditis is diagnosed more frequently in females in early or middle adolescence with a (females:males) F:M ratio of 2-5.1: 1 [4,6,7]. It results from cellular-mediated progressive destruction and fibrous replacement of the thyroid which might lead to thyromegaly or atrophy [8-11]. Furthermore, autoantibodies are formed against several thyroid-specific antigens, commonly thyroid peroxidase and thyroglobulin [12]. Hashimoto´s thyroiditis tends to be co-morbid with other non-thyroidal autoimmune diseases (NTADs) or to run in families [6]. The genetic predisposition of Hashimoto´s thyroiditis is emphasized by its aggregation in certain families and several genes are believed to be involved [6,8]. Thyroid enlargement and fatigue are the commonest presenting features, however thyroid status at onset and in long-term shows variation between different studies [3,4,6,7,13,14].

The authors aimed to study the clinical presentation, associated autoimmune diseases (ADs), and progression of Hashimoto´s thyroiditis in the Sudanese young population. No such data were previously published from Sudan.

## Methods

**Study design and setting:** this was a retrospective records review of patients who were diagnosed to have Hashimoto´s thyroiditis and presented to the Pediatric Endocrinology Unit at Gaafar Ibnauf Hospital in the period from January 2006 to November 2020. It is the only tertiary pediatric endocrinology center in the country and referrals are received from all over Sudan.

**Study population:** all records of patients less than 18-year-old at presentation, had thyroid enlargement and/or symptoms of hypo- or hyperthyroidism (with no characters suggestive of Graves´ disease) with biochemical and/or thyroid ultrasonography features of Hashimoto´s thyroiditis were collected. Those with an incomplete record, who were less than four years at presentation, had unexplained learning disabilities, no documentation for anti-thyroperoxidase (anti-TPO) and/or anti-thyroglobulin (anti-TG) testing, or coming from iodine-deficient areas (IDAs) with no biochemical or ultrasound evidence of Hashimoto´s thyroiditis were excluded.

**Data collection:** data related to demographic characters, presenting clinical and biochemical features, the coexistence of NTADs, family history of thyroid or other ADs, anthropometric measures, physical findings and workup results were collected using a predesigned data collection sheet. The thyroid gland was graded according to World Health Organization (WHO) criteria to G0 if not palpable and not visible, G1 if palpable but not visible, and G2 if visible. In addition, its consistency and surface characters on palpation were described. Patients were classified into pre-pubertal or pubertal according to their puberty status at presentation. Delayed puberty was defined as having no secondary sexual characteristics by the age of 13 in girls and 14 in boys. Thyroid function test (TFT), anti-TPO, and anti-TG were done on a commercial basis and results were considered abnormal in comparison to the laboratory reference ranges (LRR). Missing data and the last TFT were collected by contacting patients and/or their caregivers.

**Definitions:** study participants were divided into four functional subgroups according to their initial TFT. Subgroups were defined as follows: euthyroidism if thyroid-stimulating hormone (TSH) was 0.2-5 mU/L and free thyroxine (FT4) was within the LRR, sub-clinical hypothyroidism if TSH was >5 mU/L and FT4 was within the LRR, overt hypothyroidism if TSH was >10 mU/L and FT4 was below the LRR and hyperthyroidism if TSH was <0.1 mU/L and FT4 was above the LRR. The evolution of the thyroid function was assessed in those who were consistent with follow-up for at least six months.

**Statistical analysis:** the Statistical Package for the Social Sciences (SPSS) version 25, a computer-based software, was used to enter and analyze data. Results were shown in mean/median or range for numeric variables and frequencies and percentages for categorical data. The comparison between subgroup variables was carried out using analysis of variance (ANOVA) for normally distributed data, Kruskal-Wallis for skewed continuous data and Chi-square for categorical data. Significance was considered at a p-value of <0.05.

**Ethical considerations:** the study protocol was approved by the endocrine institutional review board at Sudan Childhood Diabetes Center (approval reference: ENDO-SCDC-IRB-21117).

## Results

**Demographic and presenting features:** one-hundred and twelve patients were provisionally diagnosed to have Hashimoto´s thyroiditis. Those who (n=39) were diagnosed clinically and/or based on ultrasonography findings without testing for thyroid autoantibodies and/or having incomplete records were excluded. A record of 73 patients met the inclusion criteria and were furtherly studied. The mean (range) age at presentation was 10.6±2.9 (4-17) years with a female to male ratio of 4.2: 1. Table 1 shows the descriptive data of the studied population at the diagnosis. The majority of the patients were residing in ISAs. Neck swelling and goiter were the commonest presenting complaint and rational for referral to our clinic. The median (range) duration of the illness before the presentation was five (0.5-48) months. Short stature, wasting and obesity were detected in 17.8% (n=13), 12.3% (n=9) and 11% (n=8), respectively. More than half of the patients (n=39) were pre-pubertal at presentation, two of them were having delayed puberty.

**Table 1:** clinical characteristics at presentation (n=73)

Character	Variables	Frequency, n (%)
Gender	Female/male	59 (80.8%)/14 (19.2%)
Residence iodine status	Iodine sufficient/iodine deficient	61 (83.6%)/12 (16.4%)
Presenting complaint	Neck swelling	58 (79.5%)
	Fatigue	32 (43.8%)
	Poor growth	14 (19.2%)
	Behavioral changes	11 (15.1%)
	Deteriorating school performance	8 (11%)
	Headache	5 (6.8%)
Cause of consultation	Goiter	50 (68.6%)
	Symptoms of hypothyroidism	12 (16.4%)
	Short stature	4 (5.5%)
	Symptoms of hyperthyroidism	3 (4.1%)
	Obesity	2 (2.7%)
	Abnormal thyroid function	2 (2.7%)
Weight for age	±2 SD^a^	54 (74%)
	-2 SD^a^	17 (23.3%)
	>+2 SDa	2 (2.7%)
Height for age	±2 SD^a^	60 (80.2%)
	<-2 SD^a^	13 (17.8%)
BMI^b^	>95^th^ percentile^c^	8 (11%)
	85-95^th^ percentile^c^	5 (6.8%)
	5-85^th^ percentile^c^	51 (69.9%)
	<5^th^ percentile***	9 (12.3%)
Goiter	G2^d^	57 (78.1%)
	G1^e^	3 (4.1%)
	G0^f^	13 (17.8%)
Puberty	Pre-pubertal	39 (53.4%)
	Pubertal	34 (46.6%)
Thyroid status	Overt hypothyroidism	44 (60.3%)
	Subclinical hypothyroidism	15 (20.5%)
	Euthyroid	10 (13.7%)
	Hyperthyroidism	4 (5.5%)
Thyroid USS^g^	Not done	39 (53.4%)
	Diffuse with hypoechoic areas	17 (23.3%)
	Diffuse with normal echogenicity	14 (19.2%)
	Normal size and echogenicity	1 (1.3%)
	Multinodular goiter	1 (1.3%)
	Calcification	1 (1.3%)

a : standard deviation; ^b^: body mass index; ^c^: according to Centers for Disease Control and Prevention growth chart; ^d^: goiter is visible and palpable; ^e^: goiter is palpable not visible; ^f^: goiter is neither visible nor palpable; ^g^: ultrasound scan

The thyroid gland was diffusely enlarged in the majority (82.2%, n=60) of the study population, however, it was neither visible nor palpable in 13 (17.8%) patients (Table 1). Among those with goiter (n=60), the thyroid consistency was firm in 32 (53.3%), soft in 28 (46.7%) and the thyroid gland surface was smooth in 52 (86.7%), nodular in three (5%) and multi-nodular goiter were reported in five (8.3%) of them. At presentation, 60.3% (n=44) of patients had overt hypothyroidism, 20.5% (n=15) had subclinical hypothyroidism, 13.7% (n=10) were euthyroid and 5.5% (n=4) presented with the thyrotoxicosis (Table 1). Anti-TPO was the only tested autoantibody in the majority of the study participants (72.6%, n=53). Only 27.3% (n=20) of our patients were able to test for both anti-TPO and anti-TG, 15 had elevated both anti-TPO and anti-TG and 5 had elevated anti-TG only. The most common finding on thyroid ultrasonography scan, among those who were able to do it (n=34/73), was diffuse goiter with multiple hypoechoic areas (Table 1).

There was no significant difference between the functional subgroups of the study population in the demographic features, the coexistence of NTAD, family history of autoimmune thyroid diseases (AITD), anthropometry, goiter grade or pubertal status (Table 2).

**Table 2:** comparison between functional subgroups at presentation in relation to demographic data, history and physical findings (n=73)

Character	Euthyroid (n=10)	Sub-clinical hypothyroid (n=15)	Overt hypothyroid (n=44)	Hyperthyroid (n=4)	P value
Age ± SD (years)	10.5 ± 3.2	10.6 ± 2.6	10.5 ± 3.1	11.6 ± 2.7	0.93
Gender (F/M)	8/2	14/1	33/11	4/0	0.33
Iodine status (replete/deplete)	7/3	14/1	37/7	3/1	0.46
Coexistence NTAD	0	1	5	0	0.59
FHx of AITD	5	4	18	1	0.60
Weight					
±2SD	10	14	26	4	0.44
<-2SD	0	1	16	0	
>+2SD	0	0	2	0	
Height					
±2SD	10	15	31	4	0.15
<-2SD	0	0	13	0	
BMId					
±2SD	9	13	32	2	0.64
<-2SD	1	1	6	1	
>+2SD	0	1	6	1	
Goiter					
G2	10	13	30	4	0.22
G1	0	1	2	0	
G0	0	1	12	0	
Puberty					
Pubertal	4	7	21	2	0.96
Pre-pubertal	6	8	23	2	

NTAD: non-thyroidal autoimmune disease; FHx: family history; AITD: autoimmune thyroid disease; BMI: body mass index; SD: standard deviation

**Coexistence and family history of ADs:** coexistence of NTADs were documented in 8.2% (n=6) of our cohort which included type 1 diabetes (T1D) (n=1), celiac disease (CD) (n=1), systemic lupus erythematosus (SLE) (n=1), autoimmune myopathy (n=1), myasthenia gravis (n=1), and one patient had T1D, SLE and Addison’s disease. While in two-third (n=4/6) of them, the diagnosis of Hashimoto´s thyroiditis preceded the onset of the NTAD by a median (range) of 30 (10-42) months, the other two patients were diagnosed to have Hashimoto´s thyroiditis with SLE and Hashimoto´s thyroiditis with myasthenia gravis simultaneously at presentation. ADs were reported in 29 (39.7%) families of our patients, including Hashimoto´s thyroiditis (n=22), Graves´ disease (n=4), SLE (n=1), Hashimoto´s thyroiditis/SLE (n=1), and Hashimoto´s thyroiditis/CD (n=1).

**Figure 1:**
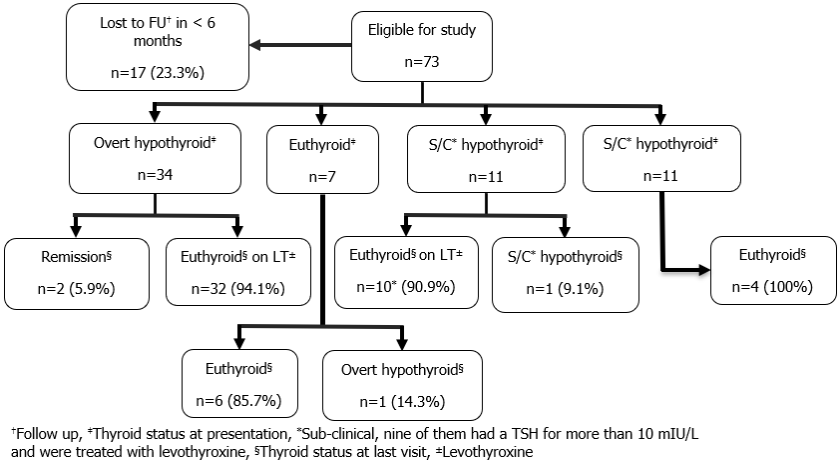
long term outcome of the study participants

**Thyroid function evolution over time:** twenty-three point three percent (23.3%, n=17) of our patients dropped out of the follow-up in less than 6 months and have been excluded from the assessment of the outcome (Figure 1). All patients with overt hypothyroidism at onset (n=34) were treated with levothyroxine, 94.1% (n=32) of them remained euthyroid while on levothyroxine for 0.5-13 years and 5.9% (n=2) stopped the therapy and had complete remission. On the other side, those who presented with hyperthyroidism (n=4) went into euthyroidism in 3.5 ± 1.1 (2-5) months with propranolol only. The majority of patients with euthyroidism at onset (n=6/7) continued to be so for 0.5-6 years duration of the follow-up and only one patient developed overt hypothyroidism five years after the diagnosis. Out of the 11 patients who presented with subclinical hypothyroidism, ten patients had a TSH of >10 mU/L and were treated with levothyroxine with an average dose of 1.31 ± 0.38 mcg/kg/d. All of them became euthyroid in three to ten months while on levothyroxine therapy and remained euthyroid for 10 months to 13 years. The remaining patient with subclinical hypothyroidism at onset had a TSH of 7.6 mU/L, was not treated with levothyroxine, and remained to have subclinical hypothyroidism during the one-year follow-up duration.

## Discussion

Hashimoto´s thyroiditis is a common thyroid disorder that we encounter in our clinic. Studying the mode of its presentation and evolution in our population will be valuable in the short and long-term management planning as well as counseling of patients and/or their caregivers. This study showed that Hashiomto´s thyroiditis is more prevalent among prepubertal females and those living in ISAs. Moreover, thyromegaly was the commonest presenting feature and the majority had impaired thyroid function. To the best of our knowledge, this is among the first studies in Africa that characterized its clinical profile among children [15].

Hashimoto´s thyroiditis, in our cohort, was significantly more prevalent in females (80%) which is consistent with different international and regional reports [3,8,13,15]. The ADs predilection towards females is well known, Hashimoto´s thyroiditis is not an exception, and that may be related to the biased X-chromosome inactivation, female sex hormone or immune system response [16-18]. The mean age of our group at the diagnosis was 10.6 ± 2.9 years which is in the line with different studies [13-15]. Sudan is a big country, many regions are known to be IDAs, therefore it was interesting to explore whether Hashimoto´s thyroiditis is more prevalent among those living in ISAs or IDAs. Eighty-three point six percent of our patients were residing in ISAs that agreed with some epidemiological studies [19,20].

Thyroid enlargement was the commonest consultation in our clinic similar to many published reports [4,15,21,22]. However, goiter was not detected in 17.8% of our cohort, non-goitrous form of Hashimoto´s thyroiditis has been described in children, consistent to a recent report from Egypt (16.8%) [15]. A significant proportion of our patients (43.8%) had fatigability and this was the commonest presenting complaint of 122 pediatric Hashimoto´s thyroiditis patients in Ibili *et al*. study [13]. It might be explained by the thyroid dysfunction, however, the autoimmune process itself is a possible contributing factor [23]. The prevalence of short stature among our cohort was 17.8% which is close to that reported in the Egyptian study (18.7%) [15].

In contrast to the literature, that most of the patients are diagnosed at their mid or late puberty, more than half of this study participants (n=37) were pre-pubertal excluding those who had delayed puberty (n=2) [8,13,24]. Furthermore, 60.3% of our series presented with overt hypothyroidism which is comparable to the Egyptian study findings (67.8%) [15]. However, many studies from developed world claimed that euthyroidism is the commonest mode of presentation [3,4,25,26]. These regional variations suggest that other factors, like environmental factors or endocrine disruptor exposure, may modulate the presentation mode of Hashimoto´s thyroiditis among children in our community [27]. Autoimmune diseases (ADs) are characterized by their tendencies to aggregate on the same patient and family. In this research, the prevalence of NTAD was 8.2% in comparison to 6.5% by Ibili *et al*. [13]. However, a higher prevalence (18.8%) has been reported in a larger young population with Hashimoto´s thyroiditis (n=553) [28]. Although some authors suggest screening for NTAD in children with AITD, the argument probably would be against it in limited-resource settings due to testing costs [3]. The high prevalence of ADs including AITDs in antecedents of Hashimoto´s thyroiditis patients in this research (39.7%), similar to 40% and 54.4% reported by Admoni and Calcaterra *et al*. respectively, emphasizes the implication of genetic factors on its pathogenesis [3,8].

This study didn´t detect significant differences between functional subgroups in demographics, presenting clinical and biochemical features. That is in agreement with Özsu *et al*. and Dündar *et al*. studies which failed to show significant differences in ages, gender, and absence of goiter between different subgroups among 106 and 78 pediatric patients with Hashimoto´s thyroiditis, respectively [22,29]. Nonetheless, later research revealed that goiter was more prevalent in those who were euthyroid at onset [29]. In addition, de Vries *et al*. study didn´t show a difference in height or body mass index (BMI) of their 112 Hashimoto´s thyroiditis patients in relation to their biochemical presentation [21]. However, contrary to our findings and those of de Vries *et al*. those with overt hypothyroidism at presentation were younger than their peers on some reports [3,4,21,30]. Furthermore, the ratio of pubertal to pre-pubertal participants was higher amongst those who were euthyroid at diagnosis [4,30]. Different sample sizes and definitions of the functional subgroups may explain the variations between these studies.

Few studies have addressed the long-term progress of childhood Hashimoto´s thyroiditis [31]. In sense of the urgent need for hormonal replacement of thyroid failure, all our patients with overt hypothyroidism at diagnosis (n=34) were treated with levothyroxine. Most of them (94.1%) required the therapy to remain euthyroid during the follow-up period which is comparable to the Gopalakrishnan *et al*. and Admoni *et al*. findings [2,3]. However, the higher rate of remission in the latter study (16%), even higher in other studies, suggests that levothyroxine is not necessary to be a lifelong therapy in hypothyroid Hashimoto´s thyroiditis patients, and it is worth reassessing the TFT while off the levothyroxine later in the course of the illness [3,32,33].

It is rare for Hashimoto´s thyroiditis to present with hyperthyroidism, 5.5% in this research compared to 3.5% on other reports, that almost always remit spontaneously in a short time with no subsequent relapse [3,30,31]. Therefore, increasing awareness about the condition among health practitioners is essential to avoid unnecessary carbimazole initiation. Most of our patients with euthyroidism at onset continued to have so for up to 6 years, however due to the risk of developing overt hypothyroidism, 14.3% in our series compared to 12.3-26% in the literature, long-term TFT monitoring is needed [3,34]. Ninety point nine percent of our patients with subclinical hypothyroidism at onset had a TSH >10 mIU/L, they were considered to have a compensated hypothyroidism and all of them required the levothyroxine to maintain euthyroidism for up to 13 years. Although there is no consensus guideline on the management of childhood and juvenile subclinical hypothyroidism so far, Crisafulli *et al*. have shown that the long-term outcome may be unfavorable in those with a TSH >10 mIU/L [35]. Moreover, an elevated TSH was found to have a possible role in the development of thyroid malignancy in patients with Hashimoto´s thyroiditis [36].

The retrospective nature of this research and the discrepancy in the follow-up duration between the participants are among the limitations. Moreover, around one-third of our cohort has been excluded due to the challenges of confirming the diagnosis. Barriers were including the cost and periodic unavailability of thyroid autoantibodies testing (around 50 US dollars in Sudan). Furthermore, in countries like Sudan where the consanguineous marriage rate is high, late-onset thyroid dyshormonogenesis cannot be ruled out without testing for thyroid autoantibodies [37].

## Conclusion

This study showed that goiter is the commonest presenting feature of Hashimoto´s thyroiditis. Therefore, testing for thyroid autoantibodies is paramount in children who present with thyroid enlargement. Unlike reports from developed countries where most of the patients are euthyroid at diagnosis, the majority of our cases had either overt or subclinical hypothyroidism requiring levothyroxine to remain euthyroid for the entire follow-up period.

### What is known about this topic

- *Hashimoto´s thyroiditis is the commonest acquired thyroid disorder in iodine-sufficient areas and more prevalent in females at mid or late puberty;*
- *Goiter and fatigability are the commonest presenting features and most of the patients in developed countries are euthyroid at the diagnosis;*
- *Some reports revealed a younger age and more prevalent goiter in those presented with overt hypothyroidism and euthyroidism, respectively.*

### What this study adds

- *More than half of our cohort were pre-pubertal;*
- *The majority of our patients presented with thyroid impairment either overt or sub-clinical hypothyroidism requiring levothyroxine to remain euthyroid during the follow-up period;*
- *Our study failed to show any significant differences in the patients´ clinical profile in relation to their presenting thyroid function status.*
